# Quantifying intra- and interlimb use during unimanual and bimanual tasks in persons with hemiparesis post-stroke

**DOI:** 10.1186/s12984-022-01020-8

**Published:** 2022-05-07

**Authors:** Susan V. Duff, Aaron Miller, Lori Quinn, Gregory Youdan, Lauri Bishop, Heather Ruthrauff, Eric Wade

**Affiliations:** 1 Department of Physical Therapy, Crean College of Health and Behavioral Sciences, Chapman University, 9401 Jeronimo Rd, Irvine, CA 92618 USA; 2 Department of Mechanical, Aerospace, and Biomedical Engineering, University of Tennessee, Knoxville, TN USA; 3 Department of Biobehavioral Sciences, Teachers College, Columbia University, New York, NY USA; 4 Department of Occupational Therapy, Children’s Hospital of Philadelphia, Philadelphia, PA USA

**Keywords:** Coupling, Interlimb use, Hemiparesis, Stroke, Wearable sensors

## Abstract

**Background:**

Individuals with hemiparesis post-stroke often have difficulty with tasks requiring upper extremity (UE) intra- and interlimb use, yet methods to quantify both are limited.

**Objective:**

To develop a quantitative yet sensitive method to identify distinct features of UE intra- and interlimb use during task performance.

**Methods:**

Twenty adults post-stroke and 20 controls wore five inertial sensors (wrists, upper arms, sternum) during 12 seated UE tasks. Three sensor modalities (acceleration, angular rate of change, orientation) were examined for three metrics (peak to peak amplitude, time, and frequency). To allow for comparison between sensor data, the resultant values were combined into one motion parameter, per sensor pair, using a novel algorithm. This motion parameter was compared in a group-by-task analysis of variance as a similarity score (0–1) between key sensor pairs: sternum to wrist, wrist to wrist, and wrist to upper arm. A use ratio (paretic/non-paretic arm) was calculated in persons post-stroke from wrist sensor data for each modality and compared to scores from the Adult Assisting Hand Assessment (Ad-AHA Stroke) and UE Fugl-Meyer (UEFM).

**Results:**

A significant group × task interaction in the similarity score was found for all key sensor pairs. Post-hoc tests between task type revealed significant differences in similarity for sensor pairs in 8/9 comparisons for controls and 3/9 comparisons for persons post stroke. The use ratio was significantly predictive of the Ad-AHA Stroke and UEFM scores for each modality.

**Conclusions:**

Our algorithm and sensor data analyses distinguished task type within and between groups and were predictive of clinical scores. Future work will assess reliability and validity of this novel metric to allow development of an easy-to-use app for clinicians.

## Introduction

Finely tuned upper extremity (UE) intra- and interlimb use is controlled through intact neural coupling [1], which requires timing of movements and sequential, rhythmic use of limb segments on one or both sides of the body [2]. This upper limb coupling enables interaction with the environment and the performance of goal-oriented tasks such as activities of daily living (ADLs). The type of tasks performed range from unimanual (single limb use), to bimanual symmetric (mirrored), to bimanual asymmetric with different motion exhibited in each limb. For persons with hemiparesis post-stroke, tasks requiring UE coupling can be difficult to execute due to limited strength, mobility, and motor control resulting in the execution of compensatory, yet functional, movement patterns [3–5]. Compensatory strategies may include increased trunk involvement during arm motion, limb disuse or asymmetry during mirrored bimanual tasks, and inefficient motion or atypical synergistic movements during task performance [6–8]. Although compensation promotes independence in everyday tasks, it can also impede recovery of intra- and interlimb use inherent in unimanual and bimanual performance [5, 9]. Determining the extent of coupling within and between the arms and how it changes with recovery and rehabilitation requires assessment measures sensitive to subtle changes in motion and task performance.

Most UE clinical assessments evaluate function of the paretic limb during unimanual tasks with limited emphasis on bimanual function [10–12]. An exception to this is the Assisting Hand Assessment (AHA) [13, 14], a tool originally designed to assess how effectively the more affected limb is used during bimanual tasks in children with unilateral UE dysfunction. The AHA has been recently adapted for use in adults post-stroke, i.e. the Adult AHA Stroke (Ad-AHA Stroke) [15]. However, as with other observation-based tools, the Ad-AHA Stroke may not be sensitive enough to detect small yet significant changes in motor behavior occurring with natural recovery or rehabilitation. A highly sensitive, objective measure requiring minimal equipment is needed to quantify intra- and interlimb use across a range of tasks and settings.

Inertial measurement units (IMU), are body-worn sensors that monitor and transmit changes in movement during the execution of everyday tasks [16]. IMU sensors have been used with individuals post-stroke and other neurological conditions, to capture the quality and quantity of motion during typical and atypical motor behaviors [16, 17]. These sensors can detect quantitative changes in movement patterns that differentiate between typical and atypical motor behavior. A challenge in using IMU sensors is that they produce derived, differential motion measures, such as linear acceleration and angular rate of change. Therefore, unlike traditional marker-based motion capture systems, raw data cannot be easily used to directly reconstruct changes in limb position. Instead, IMU data requires custom signal and data processing techniques to produce clinically relevant metrics [18].

Development of an accurate yet sensitive system using IMU data to identify distinct features of UE intra- and interlimb use is a sequential process. For our purposes, we operationalize intra- and interlimb use in regard to amplitude, time domain and frequency domain. Results of our pilot work suggest that development of a single motion parameter per sensor, using a novel algorithm, would allow comparison by task type between groups and allow for initial validation against widely used clinical measures [19]. The objectives of this current study were to: (1) evaluate the ability of sensor-derived motion parameters to distinguish between UE task type (unimanual, bimanual symmetric, and bimanual asymmetric tasks) in healthy controls; (2) evaluate the ability of motion parameters to differentiate between UE intra- and interlimb use in healthy controls and individuals post stroke; and (3) validate findings from sensor-derived motion parameters against clinical measures commonly used to assess performance in persons post-stroke, including the UE Fugl-Meyer (UEFM) and the Ad-AHA Stroke Assessments.

## Methods

### Participants

We recruited individuals post stroke and healthy-age matched controls from two clinical sites: Columbia University Irving Medical Center/Teachers College, Columbia University and Chapman University. Inclusion criteria for participants post stroke were: (1) > 1 year, post stroke; (2) ability to isolate elbow and shoulder motion in one arm; and (3) ability to perform a gross grasp or pinch, i.e. 3-jaw chuck or lateral pinch with both hands. Exclusion criteria included: (1) joint contractures > 20° at either elbow or > 45° in either shoulder; and (2) known allergies to tape or other skin sensitivities. Potential participants were recruited using flyers and referrals from existing databases. This study was approved by the Institutional Review Boards at Columbia University Irving Medical Center, Teachers College, Columbia University and Chapman University. All participants provided written informed consent.

### Clinical characteristics and demographics

All participants were assessed with the Edinburgh Inventory to determine handedness [20], the Manual Ability Measure-36 (MAM-36) to assess hand function [21], and the Jamar® Dynamometer and B&L Engineering Pinch Gauge to assess grip and pinch strength [22]. The MAM-36 rates 36 everyday tasks based on self-reported manual ability with the total score ranging from 0 to 144; higher scores indicate better-perceived manual ability. Grip, lateral pinch, and palmar pinch strength are reported as the mean of three trials. Persons post-stroke were also assessed using the UEFM to establish motor impairment based on a maximum score of 66 with a higher score indicating better motor function [10, 23, 24].

### Experimental procedure

**Fig. 1:**
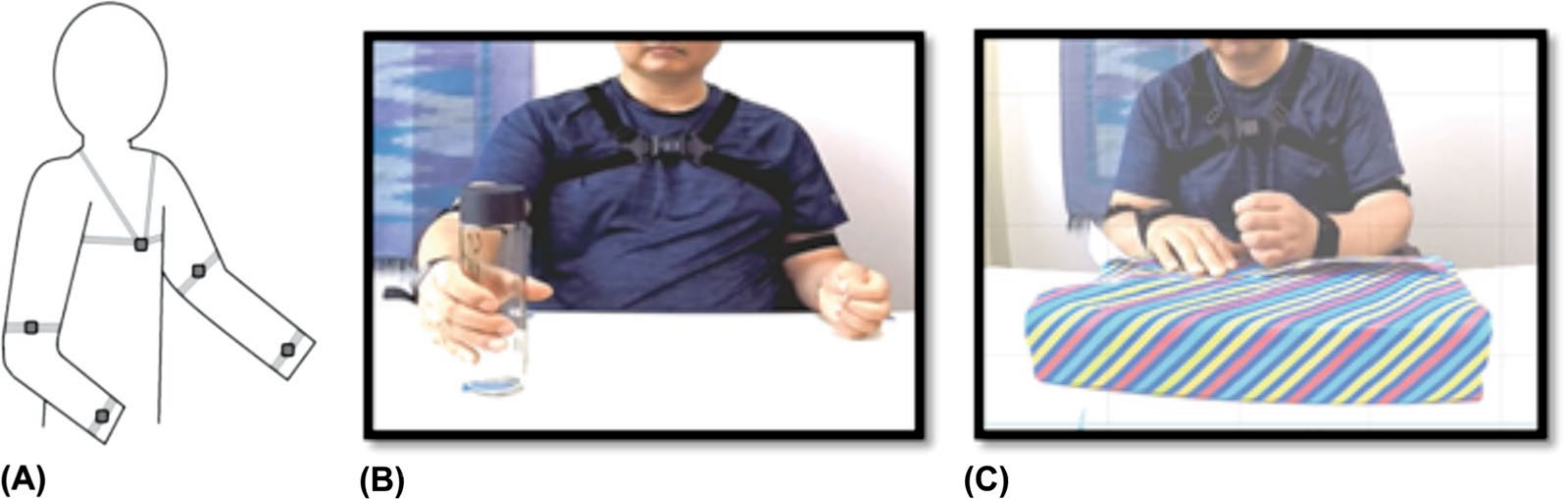
Subjects wore 5 sensors on the sternum, L/R wrist, and L/R upper arm (**A**) during performance of 6 different tasks performed twice (**B**), including reaching for a water bottle as shown here; and performance of the Adult Assisting Hand Assessment Stroke (AdAHA-Stroke), which involved unwrapping and wrapping a present (**C**)

Participants completed six UE tasks and the Ad-AHA Stroke [15] while seated and wearing five APDM Opal wearable sensors (Portland, OR). Performance was videotaped to obtain accurate start and stop times for all tasks. The sensors were secured with adjustable straps on each wrist, each upper arm, and the sternum (Fig. 1A). Each sensor recorded tri-axial acceleration, angular rate of change, and magnetic field strength at 128 Hz. Prior to collecting data during the UE tasks and Ad-AHA Stroke, one sensor was shaken in a rhythmic pattern to facilitate post–hoc synchronization of inertial and video data. The six UE tasks (see sample Fig. 1B) were counterbalanced for order and completed twice by each participant. Tasks included two unimanual (Uni) tasks (reaching for a bottle and reaching across midline for a spoon); two bimanual symmetric (BS) tasks (folding a towel and donning a hat); and two bimanual asymmetric (BA) tasks (unscrewing a bottle lid and stirring marbles in a bowl). In principle, there should be no kinematic coupling between limbs for unimanual tasks, a high level of coupling for bimanual symmetric tasks, and moderate coupling for bimanual asymmetric tasks.

Paretic limb performance in persons post-stroke was further assessed using the Ad-AHA Stroke [15], which required participants to unwrap and wrap a present (Fig. 1C). Specifically, participants were informed that the aim of the task was to use both hands in the way that felt most natural. This would allow assessment of functional performance when both hands were used together. Scoring of the Ad-AHA Stroke from videotape was done on 19 components within five categories: general usage, arm use, grasp-release, fine-motor adjustment, and coordination. Sample components included items such as initiates use, stabilizes by grip and flow in bimanual task performance. A 1–4 category rating scale was used to score the more affected limb on all 19 components: (1) does not do; (2) ineffective; (3) somewhat effective; and (4) effective. The Ad-AHA Stroke has established validity and reliability in individuals post stroke [25]. All ratings were completed by an experienced and certified rater.

### Data processing

Use of IMU data requires custom signal and data processing techniques to produce clinically relevant metrics [18]. Raw sensor data were filtered using a 3rd order Butterworth bandpass filter with 0.1 Hz and 2 Hz cutoff frequencies and then digitally de-trended to remove drift [18]. We developed a novel algorithm to allow this sensor data to be used to detect similar task types [19]. This algorithm is fully described in our prior work; here, we provide a brief description for context.

For the full algorithm, we combined three axes of data (x, y, z), from three sensor modalities (acceleration, angular rate of change, and orientation), for five sensors, using a ‘ranked similarity’ approach. Initially, data from each sensor were compared to every other sensor resulting in ten pairs. Specifically, we determined which was the leading limb for bimanual tasks and which was the moving limb for unimanual tasks based on performance of controls. The wrist 1 and upper arm 1 sensors were classified as *more active* and the wrist 2 and upper arm 2 sensors as *less active* during each task. The ten sensor pairs were: a) sternum to wrist 1 (S-W1); b) sternum to wrist 2 (S-W2); c) sternum to upper arm 1 (S-U1); d) sternum to upper arm 2 (S-U2); e) wrist 1 to wrist 2 (W1-W2); f) wrist 1 to upper arm 1 (W1-U1); g) wrist 1 to upper arm 2 (W1-U2); h) wrist 2 to upper arm 1 (W2-U1); i) wrist 2 to upper arm 2 (W2-U2); and j) upper arm 1 to upper arm 2 (U1-U2).

**Fig. 2:**
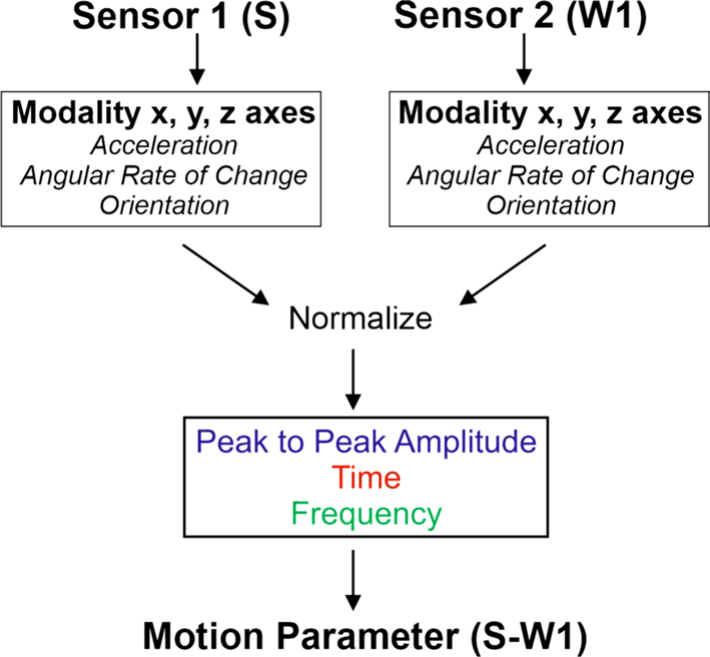
The motion parameter for each sensor pair (i.e., S-W1) was created by averaging the metrics of time, frequency and peak-to-peak amplitude of the normalized modalities (acceleration, gyroscope, magnetometer) of each axis for the two sensors

The dynamic 3D motor behaviors required further analyses of the sensor-derived data. To determine the similarity between the motion of any two segments (e.g., S to W1), data from each modality (e.g., acceleration) were compared between sensor pairs for three metrics: peak to peak amplitude, the time domain (correlation), and the frequency domain (spectral coherence). As shown in Fig. 2, for each modality (i.e., acceleration) data from the x-, y-, and z- axes of one sensor were compared to the data from the x-, y-, and z- axes of another sensor. Data from each modality was normalized separately for each participant. This resulted in comparable data for the metrics of relative amplitude, time domain similarity (correlation), and frequency domain similarity (spectral coherence) [19]. Data for the three metrics were then averaged to create a single value for comparison between each pair, for each task, titled the *motion parameter*.

**Fig. 3:**
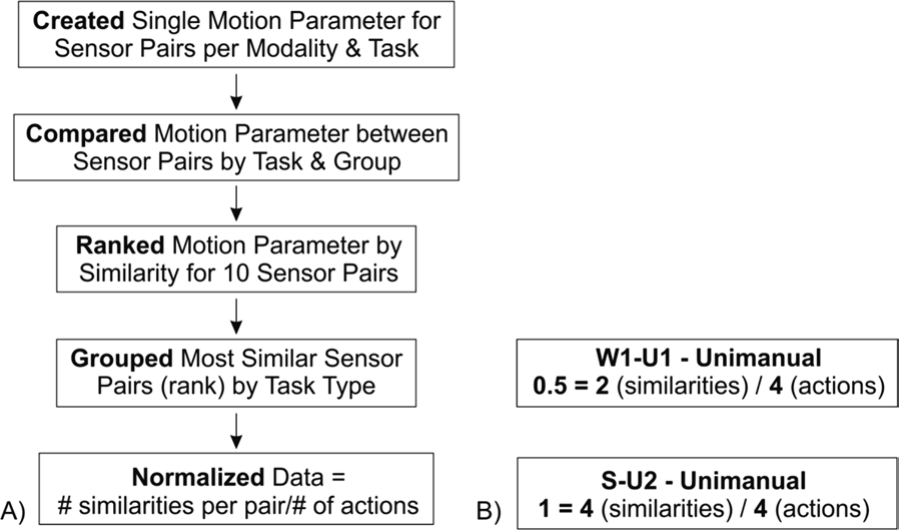
**A** Sequence of algorithm construction; and **B** two examples

The sequence for execution of our algorithm following calculation of the motion parameter(s) is shown in Fig. 3. To assess intra and interlimb coupling the motion parameters for the ten sensor pairs were ranked from highest to lowest based on the similarity value (with 1 being most similar, and 0 being least similar). Then the most similar sensor pairs by rank order, were grouped by task type (e.g., unimanual). To normalize the data for task type, we divided the number of times each sensor pair had a high similarity value by the number of actions performed. Our algorithm was based on task type and performance by controls. For example, each participant performed four unimanual tasks. We would expect a control to have a high similarity value between the sternum and inactive upper arm (S-U2) for all four iterations of unimanual tasks since both body parts are apt to move very little during task performance. This sensor comparison would result in a similarity score of 4/4 or 1. Conversely, since data from the wrist and upper arm sensors of the active limb (W1-U1) of a control may have same motion but active at different amplitudes, that sensor pair might only demonstrate similarity for two of the four task iterations resulting in a similarity score of 2/4 or 0.5. Thus, the top ranked sensor pairs per task type with the highest frequency often received scores closer to "one," while sensor pairs that were low ranking often received scores closer to "zero." The ranking was performed for each task type and metric, and the similarity values for each participant post-stroke were compared to an age-matched control. Further details of this approach may be obtained from prior work [19].

In persons post-stroke, we extracted a use ratio during performance of the Ad-AHA Stroke (present wrapping task), by comparing the wrist sensor data from the paretic arm against the non-paretic arm (paretic/non-paretic arm). This use ratio was also compared to scores obtained for the UEFM. For each sensor modality (acceleration, angular rate of change, and orientation), the total area under the raw data curve (integral) for the paretic arm was normalized by the total area under the curve (integral) for the non-paretic arm. Thus, the use ratio for each modality was compared against the AdAHA Stroke logit score and the UEFM score for each person post-stroke.

### Data analyses

**Fig. 4:**
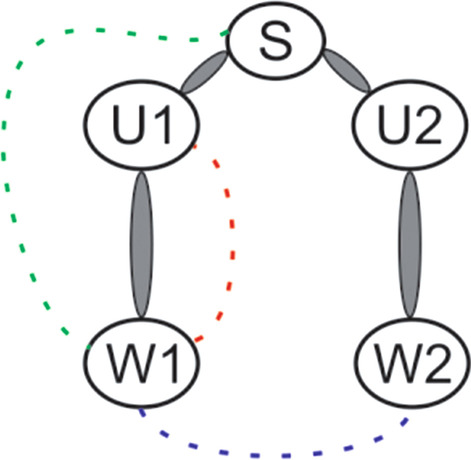
Three key sensor to sensor comparisons: S-W1 (green line), W1–W2 (blue line), and W1–U1 (red line)

Algorithm outputs for the similarity value were examined using a two-way analysis of variance (ANOVA) to assess performance in persons post-stroke vs. controls across the three task types (Uni, BS, BA). Specifically, a 2 (group) × 3 (task) ANOVA was run separately for each of the 10 sensor-to-sensor comparisons based on sensor location. We present results from three (of ten) primary sensor-to-sensor comparisons that represent the most frequently observed compensatory movement patterns for persons post-stroke during upper limb tasks [6–8]. These key sensor comparisons shown in Fig. 4, were: sternum to wrist 1 (S-W1), wrist 1 to wrist 2 (W1-W2), and wrist 1 to upper arm 1 (W1-U1) for all three task types for each group. Thus, potential compensatory patterns assessed based on sensor comparison included trunk involvement during arm motion (S-W1); limb disuse or asymmetry during bimanual tasks (W1-W2), and movement efficiency or atypical synergistic motion during any task type (W1-U1). A comparison of similarity values between task type for the three key sensor pairs among each group were further examined using a Tukey–Kramer post-hoc analysis.

The arm use ratio was compared to scores from the Ad-AHA Stroke and the UEFM in persons post-stroke, using a predictive linear regression analysis to determine the extent to which the use ratio of raw wrist sensor data for each modality extracted during task performance could predict scores on each clinical assessment.

## Results

Forty participants were recruited; 20 individuals post stroke (53.5 ± 11.5 years) and 20 age-matched controls who had not had a stroke (52.3 ± 12.5 years). Individual demographics and clinical features of participants with hemiparesis post-stroke are shown in Table 1 with means (SDs) listed for controls. All participants were included in the analyses, except for the comparison of the arm use ratio against the UEFM and Ad-AHA Stroke which only included findings from participants’ post-stroke.

**Table 1:** Participant characteristics

ID	Age (years)	Post-stroke (years)	Gender	More affected side	EI	Hand	UE-FMA	MAM	Grip (kg) LA/MA, D/ND	Lateral Pinch (kg) LA/MA, D/ND	Palmar Pinch (kg) LA/MA, D/ND
1	67	4	F	L	80	R	29	107	28/4	7.6/2.5	7.3/0.0
2	60	11	M	L	100	R	9	112	32/5	4.0/0.0	6.0/1.0
3	52	10	F	L	100	R	35	122	29/12	4.0/2.0	5.3/2.5
4	64	13	F	R	− 90	L	29	130	30/2	4.0/0.5	4.0/0.8
5	67	11	M	R	− 60	L	25	110	25/8	4.0/0.0	3.0/0.0
6	25	11	M	L	70	R	30	134	48/18	6.0/0.0	4.0/1.0
7	64	8	F	R	− 100	L	32	112	25/4	6.0/0.0	4.0/0.5
8	41	4	F	R	− 70	L	27	123	25/8	4.0/4.0	5.0/0.0
9	50	3	M	R	− 80	L	26	121	45/15	7.5/2.0	7.0/2.5
10	43	10	M	R	− 100	L	31	114	35/8	8.0/2.0	5.0/1.0
11	52	2	M	L	100	L	21	33	43/2	11.5/1.5	10.0/0.0
12	56	3	M	R	27	Am	64	121	37/26	9.6/7.6	6.0/6.3
13	66	8	M	R	− 62	L	31	130	33/8	7.9/2.7	5.8/1.3
14	67	5	M	L	90	R	35	107	32/4	9.5/2.8	5.0/0.5
15	59	2	M	R	80	R	45	123	30/18	9.0/5.8	6.8/6.0
16	40	6	M	L	78	R	44	103	40/8	8.0/6.0	6.1/1.1
17	50	16	M	R	100	R	64	130	40/35	8.4/9.1	7.0/5.8
18	42	8	F	L	100	R	42	105	23/1	6.7/2.0	4.2/0.3
19	46	11	M	L	68	R	28	99	36/6	8.5/2.3	7.0/1.3
20	58	8	F	L	100	R	35	114	36/6	5.9/1.3	4.0/1.2
HC n = 20	52.3 (12.5)	NA	5 M/15F	NA	75 (37)	18R/2Am	NA	143 (1.5)	32.3 (7.4)/ 29.3 (7.4)	6.9 (2.4)/ 6.5 (2.5)	5.6 (2.2)/ 5.2 (1.7)

*ID* identification; *HC* healthy controls; *F* female; *M* male; *EI* Edinburgh Inventory; *Hand* handedness; *R* right; *L* Left; *Am* Ambidextrous; *UE* upper extremity; *FM* Fugl-Meyer Assessment; *MAM* Manual Ability Measure; *kg* kilograms; *LA* less affected; *MA* more affected; *D* dominant; *ND* non-dominant

**Fig. 5:**
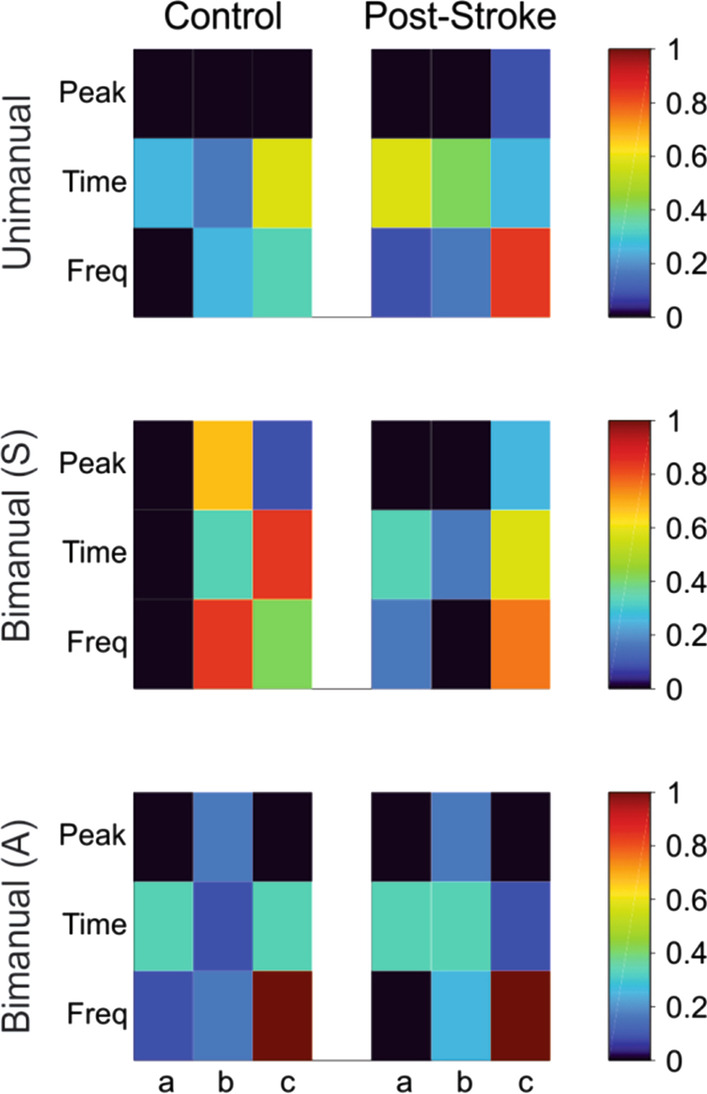
Similarity metrics set at a range of 0 (least similar) to 1 (most similar) for representative participants (Post-stroke—#19, 46 years old, UE-FMA-30; and Control #5, 49 years old) for the 3 key sensor-to-sensor comparisons by task type: Unimanual (Uni), Bimanual Asymmetric (A), and Bimanual Symmetric (S). The comparisons were: **a** sternum to wrist 1 (S–W1), **b** wrist 1 to wrist 2 (W1–W2); and **c** wrist 1 to upper arm 1 (W1–U1). Blocks in dark red indicate similarity was closer to 1.0; black blocks indicate similarity was closer to 0

**Fig. 6:**
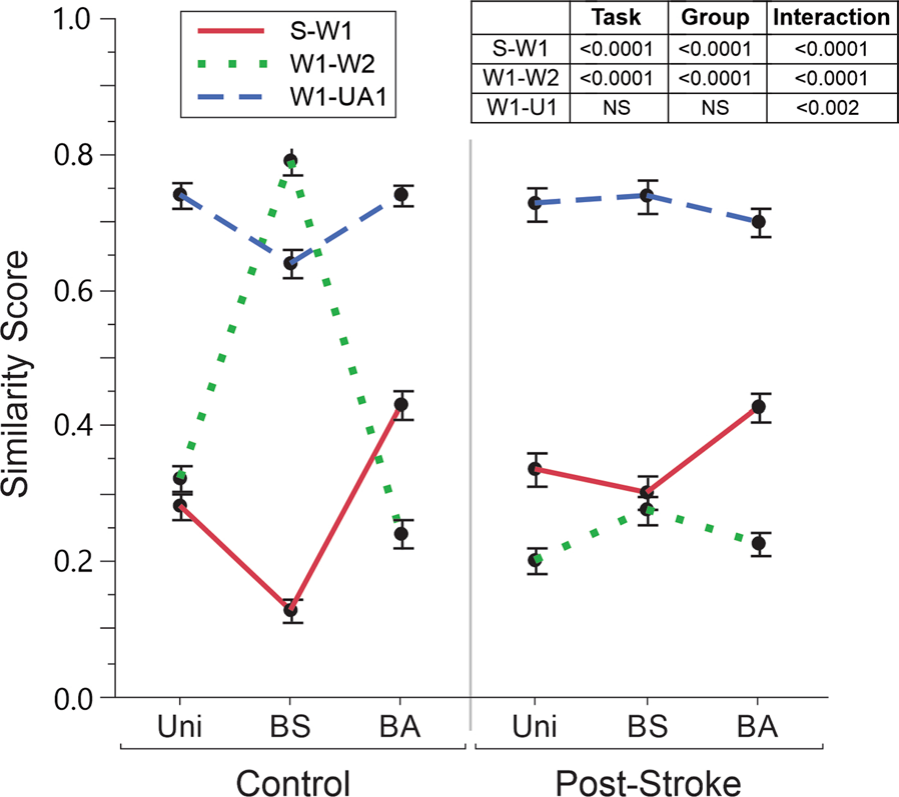
Mean with standard error of the mean (SEM) similarity metrics by group for each task type (unimanual, bimanual symmetric, and bimanual asymmetric) and the three sensor-to-sensor comparisons: Wrist to sternum (S–W1), wrist 1 to wrist 2 (W1–W1), and wrist 1 to upper arm 1 (W1–U1).

Differences in similarity value between groups (controls and persons post-stroke) across task type (Uni, BS, and BA) were found. Figure 5 depicts the similarity values for one representative participant post-stroke and one control for the three key sensor comparisons: S-W1, W1-W2, and W1-U1 across the three types of tasks. The similarity value between sensor pairs are represented by the colormap (dark red = high sensor similarity and limb coupling; dark blue = low sensor similarity and limb coupling). The relationships for group across three task types based on the similarity score for three sensor comparisons are shown in Fig. 6. As expected, we found group and task inter- and intralimb differences. Main effects for group were found for two sensor comparisons: S-W1, (F = 55.33, p < 0.0001); and W1-W2 (F = 143.28, p < 0.0001). Main effects were also found for task type for two sensor comparisons: S-W1 (F = 20.17, p < 0.0001) and W1-W2 (F = 183.23, p < 0.0001). A significant group by task interaction was found for all three sensor comparisons: S-W1 (F = 9.73, p < 0.0001); W1-W2 (F = 90.54, p < 0.0001); and W1-U1 (F = 6.22, p < 0.0001).

To further understand the significant differences noted in Fig. 6, we did post-hoc analyses of the similarity values. Among controls, the three key sensor pairs for the three task comparisons revealed significant differences for 8 out of 9 instances (Table 2). For the Uni-BS and BS-BA task comparisons: S-W1 and W1-W2 significantly differed (p < 0.0001 each); and W1-U1 significantly differed (p < 0.0004 each). For the Uni-BA tasks, the similarity values among two sensor pairs differed significantly: S-W1 (p < 0.0001) and W1-W2 (p < 0.01). For persons post-stroke, only 3 out of 9 instances were significantly different (Table 2). For Uni-BS and Uni-BA tasks, only S-W1 differed significantly (p < 0.0002 and p < 0.01 respectively), whereas, for BS-BA tasks, only W1-W2 significantly differed (p < 0.02).

**Table 2:** Findings from post-doc analyses between the three sensor pairs and three task comparisons per group

	Controls			Persons post-stroke		
	S-W1	W1-W2	W1-U1	S-W1	W1–W2	W1–U1
Uni-BS	< 0.0001	< 0.0001	< 0.0004	< 0.0002	NS	NS
Uni-BA	< 0.0001	< 0.01	NS	< 0.01	NS	NS
BS-BA	< 0.0001	< 0.0001	< 0.0004	NS	< 0.02	NS

*S* sternum; *W1* wrist 1; *W2* wrist 2; *U* upper arm; *Uni* unimanual; *BS* bimanual symmetric; *BA* bimanual asymmetric

**Fig. 7:**
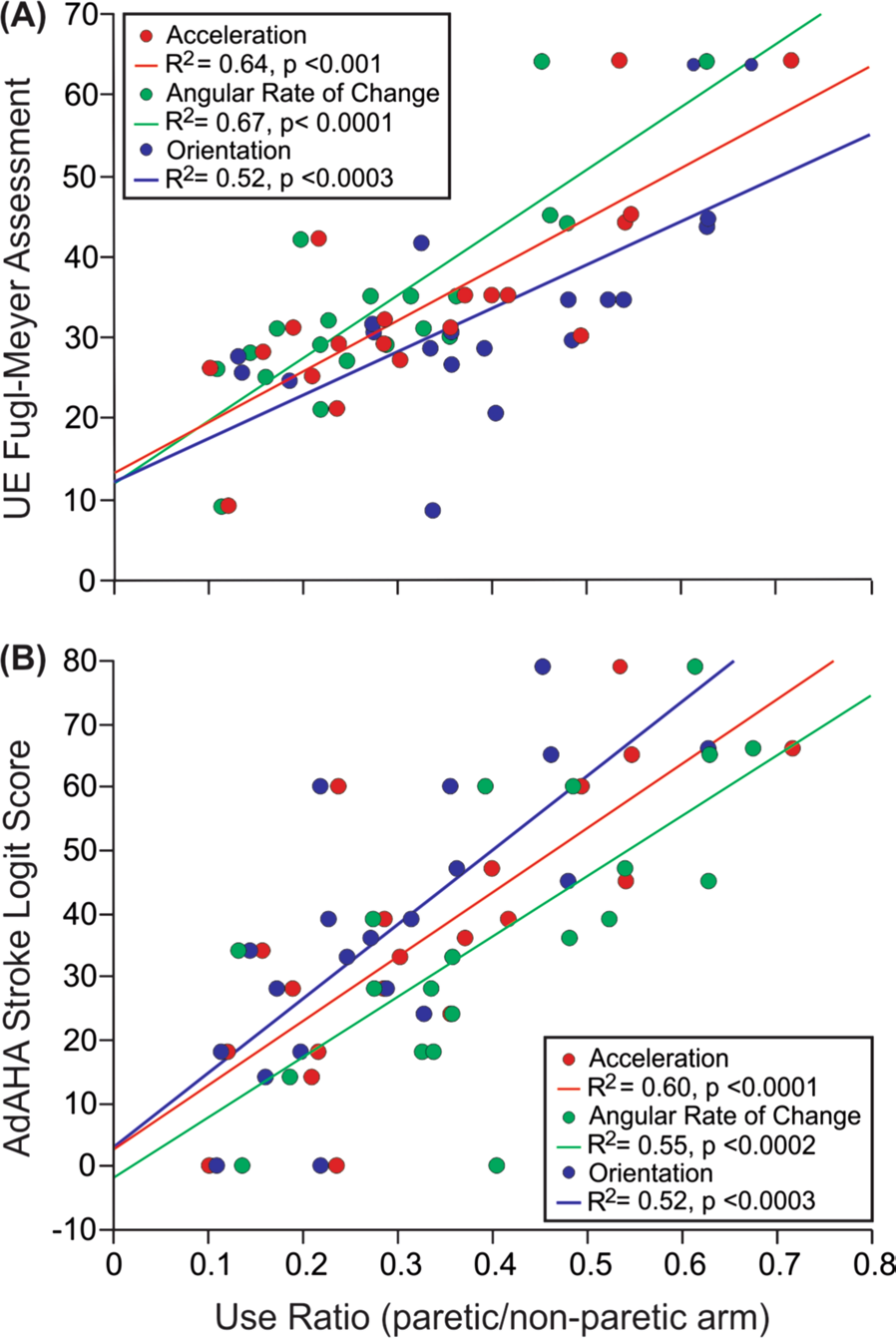
Arm Use Ratio based on the integral from wrist sensor data (paretic/non-paretic arm) for acceleration, angular rate of change, and orientation associated with **A** UE Fugl Meyer Score; and **B** Ad-AHA Stroke logit score

We separately examined the relationship between the use ratio (paretic/non-paretic arm) for all three modalities and scores on the UEFM and AdAHA Stroke. As shown in Fig. 7A the use ratio was significantly predictive of scores on the UEFM based on the integral for acceleration (R^2^ = 0.67, p < 0.0001), angular rate of change (R^2^ = 0.67, p < 0.0001), and orientation (R^2^ = 0.46, p < 0.0003). The use ratio was also predictive of scores on the Ad-AHA Stroke (Fig. 7B) based on the integral for acceleration (R^2^ = 0.60, p < 0.0001); angular rate of change (R^2^ = 0.55, p < 0.0002) and orientation (R^2^ = 0.52, p < 0.0003).

## Discussion

This study sought to quantify intra- and interlimb use during performance of unimanual and bimanual tasks via a novel algorithm comparing performance between individuals post-stroke and controls. The findings suggest that our sensor-based algorithm accurately discriminated between groups. While it strongly differentiated between Uni, BS, and BA tasks in controls, this was less clear for persons post-stroke. The key sensor-to-sensor comparisons for task type revealed strong co-operative motion of the more affected arm and trunk even during BS tasks. Lastly, the use ratio for each modality was found to be predictive of scores on the UEFM and Ad-AHA Stroke.

Temporal coupling between the limbs is essential for bimanual task performance. During symmetric tasks, one limb typically mimics the other regarding speed and movement pattern causing a mirror-like effect. Similar interlimb performance for symmetric tasks has been reported in healthy adults during bilateral pointing and lifting tasks [26]. In children and adults with hemiparesis, slower movement of the paretic limb typically slows performance of the less affected limb resulting in similar movement duration with compensatory kinematics [27, 28]. Here, we were able to detect typical performance as well as compensatory strategies by examining the similarity score between sensor pairs. During symmetric tasks, such as folding a towel or donning a hat, there were much higher similarity scores for the two wrist sensors among controls than for persons post-stroke. In persons with hemiparesis there were higher similarity scores between the more active wrist and the sternum than for controls during tasks classified as bimanual symmetric. One explanation for this result is the linking of movement between the more affected arm and trunk during task performance, a visible, kinematic strategy often used in individuals post stroke to compensate for incomplete or weak shoulder flexion or elbow extension [7, 29–31]. Further examination of findings from the three sensor modalities and metrics combined with surface electromyography (EMG) or neuroimaging may provide greater insight as to the neural underpinnings of these relationships during task performance [32]. For example, surface EMG could reveal that the amplitude and timing of muscle activation in the more affected limb is limiting interlimb coupling during bimanual symmetric task performance in persons post-stroke.

During asymmetric tasks, the role of each limb is typically differentiated yet performance of one limb may be affected by the constraints faced by the contralateral limb [33]. Many studies have used the drawer-opening task [34, 35] to examine asymmetric task performance. In healthy children and adults, the act of opening the drawer with one hand and reaching to pick up a peg with the other occurs almost simultaneously regardless of the role of each hand [27, 36]. Yet, in children and adults with hemiparesis, the actions between limbs for this task are typically more sequential [27, 33] and performance differs depending on the role of each limb. In this study, the asymmetrical tasks involved object stabilization with one hand and movement of an object with the other hand. Interestingly, for these BA tasks, the similarity scores for the three key sensor comparisons did not significantly differ between groups. Thus, the combined metrics gathered from the motor behaviors of reaching, holding, and moving for the bimanual asymmetric tasks were generally similar between limbs among both groups.

Studies examining arm use have often used wrist-based sensors to record kinematics during unimanual and bimanual tasks [27, 28, 34, 37]. The use of two sensors allows for the calculation of an arm use ratio thus the examination of changes in arm function during recovery or over the rehabilitation process. The relationship between the use ratio (based on the integral of sensor modalities) and the UEFM found in this study is consistent with existing literature [27, 38, 39], suggesting that the tasks we used are representative of functional tasks known to be sensitive to post-stroke impairment and function. Unique to this study is the finding that the arm use ratio was also predictive of Ad-AHA Stroke logit scores during the present wrapping task. Given the sensitivity of the UEFM motor domain and Ad-AHA Stroke to limb coordination, the relationship with these clinical measures provides preliminary support for the validity of this sensor-based metric as a method to examine intra- and interlimb use.

### Limitations

The features examined in this study were chosen to extract quantitative information relevant to arm use during unimanual and bimanual task performance. Yet, the specificity of the extracted features and the complexity of the algorithm may limit its widespread application without further refinement. Also, movements in monitored (e.g., lab and clinical) settings may differ from those performed in ambient settings as shown for a variety of behaviors in clinical and non-clinical populations [29]. Participants in this study engaged in data collection in a simulated setting, thus, the function of the algorithm may decrease when participants perform activities in ambient settings such as the home or community. Finally, the amount and type of arm use in persons with hemiparesis can vary even for similar bimanual tasks. While we found high similarity values within group for task type, our study only had participants perform two repetitions each for six different tasks. Studies with a larger sample size and more diverse tasks may reveal greater variability for individuals post-stroke.

## Future work

For this line of analysis to be useful in clinical research, the reliability of these sensor-based measures over multiple days must be investigated and this metric of intra- and interlimb use validated against motion capture systems. Eventual integration of such tools into clinical and community use will require a minimization of cost, either through the selection of more consumer-available devices, or through subsidization by insurance companies by demonstrating clinical efficacy. The analysis and algorithm for this study were based on engineering principles and mathematics. Upon further refinement, this program will be streamlined and packaged as an application or software program, to allow ease of use by clinicians to track the development or recovery of intra- and interlimb UE use in persons with clinical conditions.

## Conclusions

The use of five sensors during task performance allowed us to analyze intra- and interlimb use or coupling. The analyses conducted from this sensor-based assessment allowed us to differentiate within and between task type for our two groups. Our wrist-based analyses were also predictive of clinical scores on the UEFM and AdAHA-Stroke. This level of analysis can be advantageous when quantifying change in use of the more affected limb during unimanual and bimanual tasks. Our quantitative assessment did reveal the use of compensatory mechanisms during unimanual and bimanual task performance, such as incorporation of the trunk during reaching and a reduction in acceleration and movement amplitude. With additional reliability studies and validation, our algorithm-based program could quantitatively assess intra- and interlimb arm use in persons with hemiparesis which may be of value when evaluating recovery or outcomes from training studies or rehabilitation.

## Data Availability

The datasets used and /or analyzed during this study are available from the corresponding author on reasonable request.

## Abbreviations

ADL: Activities of daily living
Ad-AHA Stroke: Adult Assisting Hand Assessment Stroke
ANOVA: Analysis of variance
BA: Bimanual asymmetric
BS: Bimanual symmetric
EMG: Electromyography
Fig: Figure
Hz: Hertz
IMU: Inertial measurement units
MAM-36: Manual Ability Measure-36
SEM: Standard error of the mean
S: Sternum
SD: Standard deviation
U: Upper arm
Uni: Unimanual
UE: Upper extremity
UEFM: Upper extremity Fugl Meyer
W1: Wrist 1
W2: Wrist 2
